# Placental DNA Methylation Related to Both Infant Toenail Mercury and Adverse Neurobehavioral Outcomes

**DOI:** 10.1289/ehp.1408561

**Published:** 2015-03-06

**Authors:** Jennifer Z.J. Maccani, Devin C. Koestler, Barry Lester, E. Andrés Houseman, David A. Armstrong, Karl T. Kelsey, Carmen J. Marsit

**Affiliations:** 1 Penn State Tobacco Center of Regulatory Science, Department of Public Health Sciences, College of Medicine, Penn State University, Hershey, Pennsylvania, USA; 2 Department of Biostatistics, University of Kansas Medical Center, The University of Kansas, Kansas City, Kansas, USA; 3 Center for the Study of Children at Risk,; 4 Department of Psychiatry and Human Behavior, and; 5 Department of Pediatrics, The Warren Alpert Medical School of Brown University and Women and Infants Hospital, Providence, Rhode Island, USA; 6 College of Public Health and Human Sciences, Oregon State University, Corvallis, Oregon, USA; 7 Department of Pharmacology and Toxicology, Geisel School of Medicine at Dartmouth, Hanover, New Hampshire, USA; 8 Department of Pathology and Laboratory Medicine, and; 9 Department of Epidemiology, Brown University, Providence, Rhode Island, USA; 10 Department of Epidemiology, Geisel School of Medicine at Dartmouth, Hanover, New Hampshire, USA

## Abstract

**Background:**

Prenatal mercury (Hg) exposure is associated with adverse child neurobehavioral outcomes. Because Hg can interfere with placental functioning and cross the placenta to target the fetal brain, prenatal Hg exposure can inhibit fetal growth and development directly and indirectly.

**Objectives:**

We examined potential associations between prenatal Hg exposure assessed through infant toenail Hg, placental DNA methylation changes, and newborn neurobehavioral outcomes.

**Methods:**

The methylation status of > 485,000 CpG loci was interrogated in 192 placental samples using Illumina’s Infinium HumanMethylation450 BeadArray. Hg concentrations were analyzed in toenail clippings from a subset of 41 infants; neurobehavior was assessed using the NICU Network Neurobehavioral Scales (NNNS) in an independent subset of 151 infants.

**Results:**

We identified 339 loci with an average methylation difference > 0.125 between any two toenail Hg tertiles. Variation among these loci was subsequently found to be associated with a high-risk neurodevelopmental profile (omnibus *p*-value = 0.007) characterized by the NNNS. Ten loci had *p* < 0.01 for the association between methylation and the high-risk NNNS profile. Six of 10 loci reside in the *EMID2* gene and were hypomethylated in the 16 high-risk profile infants’ placentas. Methylation at these loci was moderately correlated (correlation coefficients range, –0.33 to –0.45) with EMID2 expression.

**Conclusions:**

*EMID2* hypomethylation may represent a novel mechanism linking *in utero* Hg exposure and adverse infant neurobehavioral outcomes.

**Citation:**

Maccani JZ, Koestler DC, Lester B, Houseman EA, Armstrong DA, Kelsey KT, Marsit CJ. 2015. Placental DNA methylation related to both infant toenail mercury and adverse neurobehavioral outcomes. Environ Health Perspect 123:723–729; http://dx.doi.org/10.1289/ehp.1408561

## Introduction

Multiple studies have found associations between *in utero*, childhood, or early adulthood mercury (Hg) exposure and later neurologic and psychological impairment. One of the most cited is a study of Faroe Islands children exposed to Hg predominantly through a seafood-heavy diet, showing adverse neurobehavioral outcomes at 7 and 14 years of age (Grandjean et al. 1997). Early-life Hg exposure is associated with neurodevelopmental deficits (Counter and Buchanan 2004), including reduced newborn cerebellum size (Cace et al. 2011), adverse behavioral outcomes (Gao et al. 2007), central nervous system damage (Choi 1989), poor psychomotor development (Llop et al. 2012), cognitive developmental delays (Freire et al. 2010), and later-life effects (Rice 1996), including increased diabetes susceptibility (He et al. 2013).

The placenta is crucial in regulating fetal growth and development, including neurodevelopment (Lester and Padbury 2009; O’Keeffe and Kenny 2014). *In utero* environmental toxicant exposures may disrupt placental function, affecting growth factor and hormone production and detoxification activity (Maccani and Marsit 2009). Toxicants may interfere with placental function through epigenetic alterations, including changes in normal placental DNA methylation patterns (Burris et al. 2012; Suter et al. 2011; Wilhelm-Benartzi et al. 2012), which control the expression of genes involved in key placental cellular processes. Abnormal methylation alterations may have serious consequences for placental growth and functioning and, in turn, for developing infants’ health.

Hg crosses the placenta (Ilbäck et al. 1991; National Research Council 2000; Yang et al. 1997) and also accumulates within the placenta, where methylmercury (MeHg) concentrations can be double those of maternal blood (Ask et al. 2002) and disrupt placental functioning (Boadi et al. 1992). A common exposure source is fish consumption (Davidson et al. 2004), although occupational exposures and maternal dental amalgams with inorganic Hg (Davidson et al. 2004; Takahashi et al. 2001) can also increase placental Hg. A single amalgam restoration is associated with a 3- to 6-fold increase in placental Hg (Takahashi et al. 2001).

MeHg exposure has been associated with *SEPP1* hypomethylation in adult blood (Goodrich et al. 2013). *SEPP1* encodes a selenoprotein potentially involved in Hg toxicity protection (Goodrich et al. 2011), suggesting that methylation may be exposure-responsive. Although SEPP1 is expressed and active in the placenta (Kasik and Rice 1995), there have been no examinations of *SEPP1* methylation or its relationship to Hg in the placenta. The placenta is active during development, and variation in placental methylation at various genes has been associated with fetal growth and development and neurobehavior (Banister et al. 2011; Bromer et al. 2012; Filiberto et al. 2011; Marsit et al. 2012a, 2012b; Wilhelm-Benartzi et al. 2012). Thus, Hg-associated placental alterations may mediate exposure-associated neurobehavioral outcomes, even at exposure levels commonly identified in the population. Previous studies (He et al. 2013; Hinners et al. 2012; Wickre et al. 2004; Xun et al. 2013) have assessed toenail Hg for integrated exposure estimates. We hypothesized that prenatal Hg exposure, assessed through infant toenail Hg, is associated with altered placental methylation patterns that are, in turn, associated with adverse infant neurobehavioral outcomes.

## Methods

*Study design*. This sample included 192 infants with placental specimens from the Rhode Island Child Health Study (RICHS), a birth cohort of nonpathologic term pregnancies delivered at Women and Infants’ Hospital in Providence, Rhode Island. Participants underwent an informed consent process approved by the Institutional Review Boards of Women and Infants’ Hospital and Dartmouth College. Eligible infants were born at ≥ 37 weeks gestation, and small- and large-for-gestational-age (SGA and LGA) infants were oversampled. By definition, SGA infants weigh ≤ 10th percentile for their gestational age; 6 of 41 (14.6%) infants in the Hg subcohort and 36 of 151 (23.8%) infants in the NNNS (NICU Network Neurobehavioral Scales) subcohort had a birthweight percentile ≤ 10%. By definition, LGA infants weigh ≥ 90th percentile for their gestational age; 14 of 41 (34.1%) infants in the Hg subcohort and 45 of 151 (29.8%) infants in the NNNS subcohort had a birth weight percentile ≥ 90%. This analysis included 41 samples with Hg data and an independent subcohort of 151 samples with neurobehavioral assessments. Within 2 hr of birth, full-thickness sections were taken from the maternal side of the placenta and 2 cm from the umbilical cord-insertion site, free of maternal decidua. These sections were immediately placed in RNAlaterTM (AM7020; Applied Biosystems Inc.). Following ≥ 72 hr at 4°C, samples were blotted dry, snap-frozen in liquid nitrogen, homogenized via pulverization and stored at –80°C until analysis. Infants were examined with a newborn neurobehavioral assessment, the NNNS (Lester and Tronick 2004), after 24 hr of life, but before hospital discharge. Examinations were performed from 24 to 96 hr following birth.

*Exposure assessment*. First toenail clippings from all toes were requested from mothers as well as infants following discharge, and were available for 41 of 192 infants. Parents were asked to collect their own and their children’s toenail clippings and mail back toenail clippings in provided envelopes. Average time from birth to collection was 2.8 months (range, 0.3–7.1 months). Micrograms Hg per gram of toenail were analyzed (Rees et al. 2007) in the Dartmouth Trace Element Analysis laboratory. Within batches, samples below the limit of detection limit (LOD) were assigned a value half the lowest observed Hg value in that batch. Average LOD across batches was 0.382 μg/g; 26 samples were below LOD.

*DNA extraction and modification*. DNA was extracted, quantified, and bisulfite modified via QIAmp DNA Mini Kit (51304; Qiagen), ND-1000 spectrophotometer (NanoDrop) and EZ DNA Methylation Kit (D5008; Zymo Research).

*Methylation profiling*. Placental methylation was assessed at the University of Minnesota Genomics Center via Illumina Infinium HumanMethylation450 BeadArray (Illumina). Samples were randomized across batches stratified by birth weight group and sex. β-values—the ratio of fluorescent signals from methylated (M) and unmethylated (U) alleles—were used as the measure of methylation status at each locus, where β = Max(M,0)/[Max(M,0) + Max(U,0) + 100]. β-values ranged from 0 (no methylation) to 1 (complete methylation). Array quality assurance was assessed; poor-performing loci, X- and Y-linked loci, and SNP (single nucleotide polymorphism)–associated loci were removed (Banister et al. 2011), yielding 384,474 loci for 192 infants.

**Figure 1:**
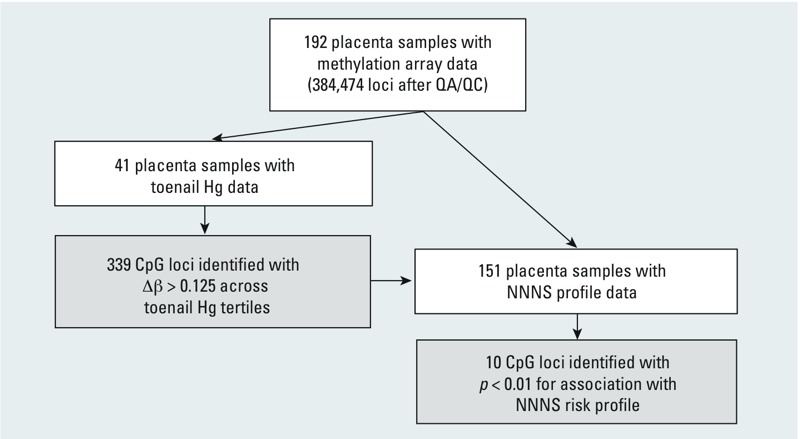
Analysis strategy: 192 placental samples were arrayed on a HumanMethylation450 BeadArray. Following quality assurance, sex-linked and SNP-associated loci were removed. Forty-one samples with Hg data were analyzed for Hg-associated placental methylation differences; 339 loci with methylation differences between Hg tertiles > 0.125 were analyzed for associations with high-risk NNNS profile in an independent set of 151 samples with NNNS data.

*Statistical analysis*. Figure 1 presents our analysis strategy. Before analysis, we assured random sample distribution across batches by Hg tertile and neurobehavioral profile; there were no associations between Hg exposure tertile and the chip or plate on which the placenta DNA sample was arrayed (*p* > 0.05). Methylation data were adjusted for plate effects via ComBat (Johnson et al. 2007), which performs effectively compared with competing adjustment methodologies. Effectiveness of this adjustment was assessed using principal components analysis and assuring no association between plate or chip and the top three principal components (all *p* > 0.05). In 41 infants with Hg data, the omnibus association between Hg tertile and methylation over 384,474 loci was tested via permutation test (Westfall and Young 1993), involving 384,474 linear regression models, one per locus, each permuted 1,000 times and controlled for maternal age (in years), birth weight percentile (continuous), delivery method (vaginal or cesarean section), and infant sex. Minimum *p*-value (over individual regression models for 384,474 loci) was used as a test statistic. Its null distribution was obtained by 1,000 draws from the permutation distribution obtained by permuting infant toenail Hg with respect to methylation and putative confounders. To avoid assuming linear response, to allow capture of relationships at the highest exposures, and to limit bias due to detection limits, we used tertiles in all analyses (Kuan et al. 2010). Individual, locus-specific *p*-values for Hg tertile were computed via standard *F*-test for H0:β1 = β2 = 0, where coefficients β1 and β2 correspond to nonreferent tertiles. Δβ-values were calculated as the difference in mean β-values between any tertile pairs. To balance sensitivity (i.e., the need to identify a comprehensive list of loci) and specificity (i.e., the need to limit false discovery), we limited the analysis of methylation and the high-risk neurobehavioral outcome to loci with Δβ > 0.125 for at least one pair of Hg tertiles.

Similar to latent profile analyses described for NNNS scores (Liu et al. 2010), mutually exclusive neurobehavioral profiles based on 13 NNNS scores were defined using recursively partitioned mixture modeling (Lesseur et al. 2013). From this analysis, seven profiles were identified, with one profile demonstrating similar attributes to that described as high-risk by Liu et al. (2010). We defined these infants as “high-risk” compared with all other infants in further analyses. Infants in this high-risk group demonstrated poorer quality of movement, poorer self-regulation, increased signs of physiologic stress and abstinence, greater excitability and the need for additional techniques for handling the infant to change state. For details on each of the summary scores between the high-risk group and other infants, see Supplemental Material, Table S1. For loci with greatest methylation differences by Hg level, we estimated the null multivariate distribution of test statistics via permutation distribution (controlled for potential confounders above) to investigate associations with the high-risk infant neurobehavioral profile in an independent sample (*n* = 151) of infants from the same study population, for whom NNNS data were available. Socioeconomic status (measured by maternal education) was examined as a possible confounder; no significant associations were identified between Hg or NNNS profile and maternal education (*p* = 0.37 and *p* = 0.70, respectively; chi-square tests), so these were not included in final models for parsimony.

Heatmaps were created in R (R Core Team 2014), using a Euclidean distance measure, to cluster placenta samples and loci based on methylation of 339 Hg-associated loci.

*Gene expression*. Total RNA was extracted via RNeasy Mini Kit (Qiagen), quantified via Nanodrop 2000 (ThermoFisher Scientific), aliquoted, and stored at –80C. Expression analysis was performed via CFX Connect Real-Time PCR Detection System (BioRad). First-strand reactions were performed in triplicate with BioRad iScript cDNA Synthesis Kit and qPCR (quantitative polymerase chain reaction) reaction with BioRad iQ SYBER Green Supermix. The sample with lowest expression served as a reference sample for delta-delta-C_t_ normalization. *EMID2* and *SDHA* expression were measured using primers: *EMID2:* forward 5´-TTTC​AGCC​TTGG​ACTT​AGCG​A, reverse 5´-GCCA​AAAT​CCTG​T CCTT​GTCA, S*DHA*: forward 5´-TGCT​CAGT​ATCC​AGTA​GTGG​A, reverse 5´-TTCT​CTTA​CCTG​TGCT​GCAA.

## Results

Table 1 describes the two study groups (infants with toenail Hg data, *n* = 41; and infants with NNNS data, *n* = 151). All infants were born at ≥ 37 weeks gestation, as required for the parent study. There is oversampling for SGA and LGA infants. Children in the two study groups were generally similar with regard to maternal age, infant sex, birth weight, or gestation time. No mothers of Hg-subcohort infants reported smoking. Among NNNS-subcohort infants, there were higher percentages of non-Caucasian mothers and cesarean section births than in the Hg-subcohort infants. Low (referent) Hg tertile ranged from 0.005 μg/g to 0.031 μg/g; medium, 0.032 μg/g to 0.076 μg/g; high, 0.077 μg/g to 0.425 μg/g. These values fall largely within a toenail Hg reference range of 0.07–0.38 μg/g derived from 130 healthy volunteers in a French study (Goullé et al. 2009). Within the Hg subcohort, there were more male infants within the medium tertile, and birth weights were higher amongst this tertile; thus, these variables were included in all models.

**Table 1:** Study population demographics.

Variable	Subset 1: Infants with toenail Hg data (*n *= 41)				Subset 2: Infants with NNNS data (*n *= 151)		
	Low Hg tertile (*n *= 14)	Medium Hg tertile (*n *= 13)	High Hg tertile (*n *= 14)	*p*-Value	Non-high-risk profile (*n *= 135)	High-risk profile (*n *= 16)	*p*-Value
Infant sex
Female [*n* (%)]	8 (57.1)	3 (23.1)	10 (71.4)	0.037	65 (48.1)	12 (75.0)	0.08
Male [*n* (%)]	6 (42.9)	10 (76.9)	4 (28.6)		70 (51.9)	4 (25.0)
Maternal age (years)
Mean ± SD	31.9 ± 3.1	32.8 ± 4.4	31.4 ± 3.3	0.76	28.4 ± 6.0	26.8 ± 6.1	0.33
Median (range)	32.5 (26–39)	33 (23–39)	30 (26–38)		29 (18–40)	26.5 (18–38)
Tobacco use during pregnancy^*a*^
Yes [*n* (%)]	0 (0)	0 (0)	0 (0)	NA	7 (5.2)	2 (12.5)	0.55
No [*n* (%)]	14 (100)	13 (100)	14 (100)		126 (93.3)	14 (87.5)
Birth weight (g)
Mean ± SD	3647.5 ± 628.2	3978.4 ±473.3	3175.7 ± 524.4	0.046	3462.9 ± 737.7	3443.8 ± 779.6	0.93
Median (range)	3,740 (2,280–4,465)	4,185 (3,035–4,530)	3,230 (2,160–4,090)		3,415 (1,705–5,465)	3,385 (2,370–4,570)
Gestational age (weeks)
Mean ± SD	39.8 ± 1.0	39.5 ± 1.0	39.5 ± 1.3	0.53	39.2 ± 1.1	39.7 ± 1.3	0.44
Median (Range)	40 (37.4–41.3)	39.7 (37.3–41.1)	39.8 (37.1–41.1)		39.3 (37–41.9)	39.5 (37–41.3)
Maternal ethnicity
Caucasian [*n* (%)]	13 (92.9)	13 (100)	11 (78.6)	0.53	99 (73.3)	10 (62.5)	0.62
Non-Caucasian [*n* (%)]	1 (7.1)	0 (0)	3 (21.4)		36 (26.7)	6 (37.5)
Cesarean section delivery
Yes [*n* (%)]	8 (57.1)	9 (69.2)	10 (71.4)	0.69	71 (52.6)	7 (43.8)	0.69
No [*n* (%)]	6 (42.9)	4 (30.8)	4 (28.6)		64 (47.4)	9 (56.3)
Recreational drug use during pregnancy
Yes [*n* (%)]	0 (0)	0 (0)	1 (7.1)	0.37	3 (2.2)	1 (6.3)	0.9
No [*n* (%)]	14 (100)	13 (100)	13 (92.9)		132 (97.8)	15 (93.8)
Maternal education^*b*^
High school graduate/equivalent or less [*n* (%)]	2 (14.3)	0 (0)	1 (7.1)	0.37	49 (36.3)	5 (31.3)	0.79
Junior college graduate/equivalent or greater [*n* (%)]	12 (85.7)	13 (100)	12 (85.7)		86 (63.7)	11 (68.8)	
NA, not applicable.^***a***^One sample with Hg data missing smoking data. ^***b***^One sample with Hg data missing education data.							

**Figure 2:**
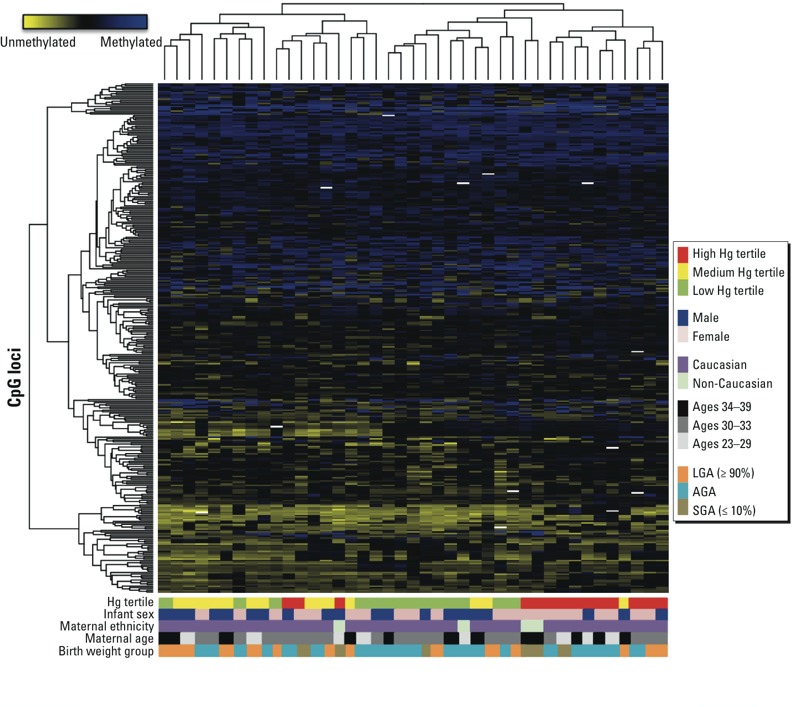
Heat map demonstrating Hg tertile differences > 0.125. Placental samples in columns; 339 loci in rows. Methylation β-values indicated by key at top left. Below figure, color bars indicate Hg tertiles (green, low tertile; yellow, medium; red, high), infant sex (blue, males; pink, females), maternal ethnicity (purple, Caucasian; green, non-Caucasian), maternal age tertiles (light gray, 23–29 years; dark gray, 30–33 years; black, 34–39 years), birth weight group [orange, LGA (≥ 90%); teal, appropriate-for-gestational-age (AGA); olive, SGA (≤ 10%)].

Mean methylation β-values were calculated for each locus by Hg tertile. Placental methylation epigenome-wide was associated with Hg (omnibus *p* = 0.017). At 339 loci, methylation differed by > 0.125 between tertiles (Figure 2; see also Supplemental Material, Table S2); generally, samples clustered by Hg and sex, but not by maternal ethnicity, maternal age, or birthweight group. Mean β-values increased monotonically with increasing Hg tertiles for 79 loci, 34 loci had a monotonic decrease with increasing tertiles, and 226 loci had a non-monotonic relationship across tertiles.

We performed supervised clustering of samples with NNNS profiles using 339 Hg-associated loci (see Supplemental Material, Figure S1), but observed no obvious clustering pattern of high-risk neurobehavioral profile. Thus, we examined individual association of loci with high-risk profile using a series of linear models; comparison of the distribution of *p*-values obtained from these models to a null distribution determined by permutation suggested that some degree of variability in risk for high-risk neurobehavioral profile membership could be attributed to methylation variation at these loci (omnibus *p* = 0.007). See Supplemental Material, Table S2, for profiles of the results of individual models. Ten loci (Table 2) residing in *CPLX1, TTC23*, and *EMID2* were associated with high-risk profile (*p* < 0.01). Six of 10 reside in a CpG island within *EMID2*, the only gene with multiple loci associated with high-risk profile within those loci at *p* < 0.01.

**Table 2:** Ten loci associated with infant toenail Hg tertile (omnibus *p *= 0.017 and Δβ > 0.125 between any two Hg tertiles) and high-risk NNNS profile (*p *< 0.01).

Illumina CpG designation	Genomic position	Relation to CpG island	Gene symbol	*p*-Value for high-risk NNNS profile
cg13267931	Chr 7: 101006308	Island	*EMID2*	8.25 × 10^–6^
cg14175932	Chr 14: 23018807^*a*^			2.84 × 10^–5^
cg27179533	Chr 7: 101006052	Island	*EMID2*	5.46 × 10^–5^
cg14874750	Chr 7: 101006063	Island	*EMID2*	6.06 × 10^–5^
cg23424003	Chr 7: 101006035	Island	*EMID2*	7.30 × 10^–5^
cg27528510	Chr 7: 101006058	Island	*EMID2*	9.00 × 10^–5^
cg14048874	Chr 7: 101006573	Island	*EMID2*	0.0023
cg17128947	Chr 4: 779480	Island	*CPLX1*	0.0054
cg25385940	Chr 15: 99789637	N Shore^*b*^	*TTC23*	0.0059
cg10470368	Chr 11: 64146517^*a*^			0.0075
Chr, chromosome. ^***a***^According to Illumina array annotation, these loci are not located within an annotated CpG region and are not associated with any gene.^*** b***^The north shore of a CpG island is defined as the region just upstream (5’) of the CpG island region.				

**Figure 3:**
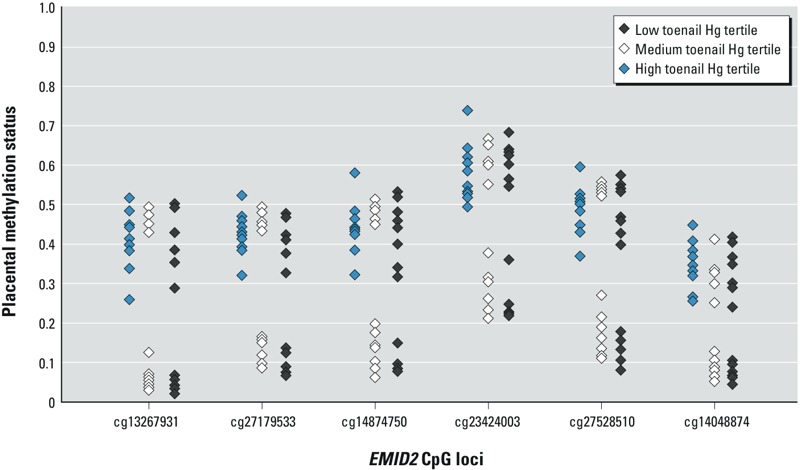
Plot of six Hg- and high-risk profile–associated *EMID2* loci in 41 samples with Hg data by tertile. *y*-Axis represents *EMID2* methylation β-value. Loci in order of appearance (+ strand, 5’ to 3’).

Four of six loci are within 200 bases of *EMID2*’s transcription start site: cg13267931 is in the 5´ untranslated region upstream of the first exon, and cg14048874 in the gene body. Figure 3 illustrates their methylation by Hg tertile. In general, those infants in the highest tertile of exposure demonstrated the highest extent of methylation at each of the CpGs present on the array.

**Figure 4:**
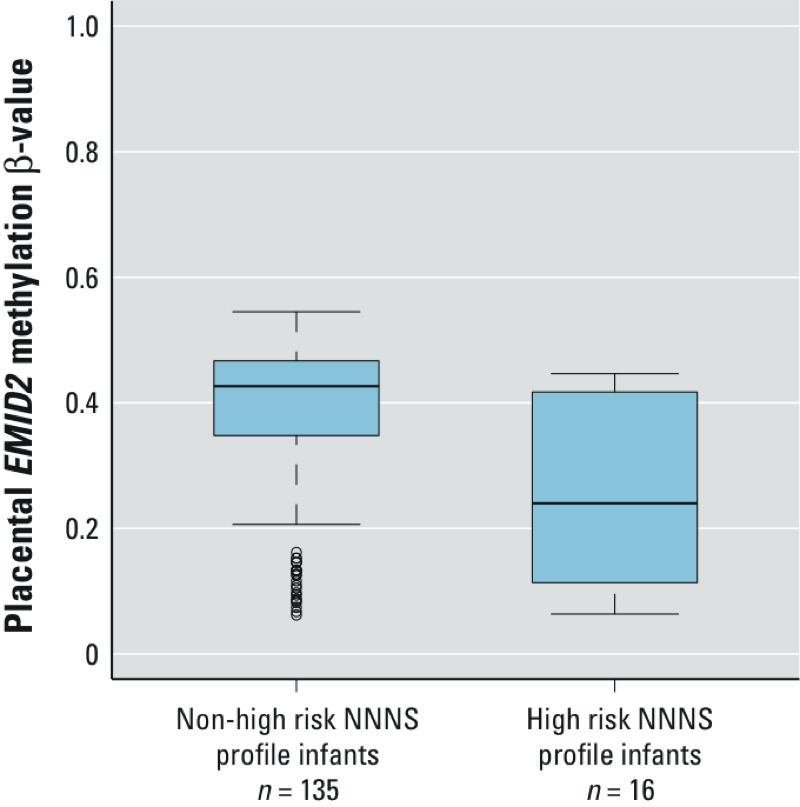
Average β-value across six *EMID2* loci associated with Hg and high-risk NNNS profile in an independent subset of 151 infants. Boxes extend from 25th to 75th percentile, horizontal bars represent medians, and whiskers extend 1.5 times the length of the interquartile range above and below the 75th and 25th percentiles, respectively. Outliers are represented as points.

We then examined the average extent of methylation across all of the *EMID2* loci in the NNNS subset, comparing those infants in the high-risk and non–high-risk groups. As shown in Figure 4, those in the high-risk group demonstrated hypomethylation of this gene. qRT-PCR in a subset of samples revealed moderate correlations between placental methylation at these loci and *EMID2* gene expression, with correlation coefficients for individual CpG loci and expression ranging from –0.33 to –0.45 (see Supplemental Material, Figure S2).

## Discussion

Placental methylation patterns were associated with infant toenail Hg and a potential high-risk infant neurobehavioral profile in our study population. Many of the 339 loci with greatest differences by Hg (see Supplemental Material, Table S2) reside in neurodevelopment-, neurogenesis- and behavior-related genes based on mutant or knockout gene studies in animal models, gene expression and knockdown studies, as well as whole genome and/or *in silico* studies (Barreto-Valer et al. 2013; Glynn et al. 2007; Heinrich et al. 2012; Ju et al. 2014; Kivimäe et al. 2011; Konno et al. 2012; Kremerskothen et al. 2002; Larsson et al. 2008; Morimura et al. 2006; Porro et al. 2010; Shimizu et al. 2010; Silver et al. 2012). Some have been associated with neurodevelopmental disorders: schizophrenia (*DIXDC1, ARVCF, MAGI2, ZIC2*) (Bradshaw and Porteous 2012; Chen et al. 2005; Sim et al. 2012), ADHD (attention deficit/hyperactivity disorder) (*TCERG1L*) (Neale et al. 2010), movement disorders (*NOL3, TP53INP2*) (Bennetts et al. 2007; Russell et al. 2012), Huntington’s disease (*H2AFY2*, *AGPAT1*) (Cong et al. 2012; Hu et al. 2011), Parkinson’s disease (*LMX1B*) (Tian et al. 2012), and autism (*PLXNA4, WNT2*) (Kalkman 2012; Suda et al. 2011). Others have been associated with diabetes (*ZBED3*) (Ohshige et al. 2011), asthma (*EMID2*) (Pasaje et al. 2011), and cancer (*FBXO3, HOOK2, MT2A, EIF3E, RPH3AL, PTRF, MT1M, STK32A*) (Cha et al. 2011; Krzeslak et al. 2013; Liu et al. 2012; Mao et al. 2012; Shimada et al. 2005).

Because of previously reported links between Hg and neurodevelopmental deficits and numerous Hg-variable genes involved in neurodevelopment, we examined these loci for associations with a high-risk newborn neurobehavioral profile defined by the NNNS, which are associated with later-life behavioral outcomes (Lester and Tronick 2004; Liu et al. 2010). In this analysis, 16 infants were observed to have a high-risk NNNS profile. We used a latent profile methodology to account for correlations between these scales and reduce data dimensionality. Liu et al. (2010) reported associations of such profiles with later-childhood outcomes: acute medical and behavior problems, school readiness, and IQ through 4.5 years of age. Of 339 loci, 10 (Table 2) were associated with a high-risk profile (*p* < 0.01) similar to that of Liu et al. (2010); 6 of 10 resided in the *EMID2* promoter.

Although *EMID2*’s placental function is unknown, its genetic variation has been associated with aspirin-sensitive asthma (Pasaje et al. 2011), and with hearing and vision side effects of the antidepressant citalopram (Adkins et al. 2012). EMID2 (or COL26A1) contains an emilin and two collagen domains primarily expressed in testes and ovary (Sato et al. 2002). *EMID2* is linked to a sonic hedgehog (*SHH*) enhancer adoption mutation, where an *EMID2* enhancer drives ectopic *SHH* expression (Lettice et al. 2011), although the loci identified are not located within that enhancer element. Future investigation is warranted to define *EMID2’s* placental role and how its modulation can impact neurodevelopment. It may be of interest to explore its role in *SHH,* which plays roles in neural tube patterning and cell survival (Ho and Scott 2002; McCarthy and Argraves 2003).

Interestingly, this potential risk neurobehavioral profile was associated with *EMID2* hypomethylation in low- and medium-Hg tertiles, with greatest hypomethylation in the mid-range group. This suggests a nonmonotonic and potentially complicated relationship between exposure, methylation, and outcome. We were limited in our ability to address these relationships in the same individuals. In addition, as we were making use of infant toenail samples, a large proportion were below the limits of detection for the assay, so extrapolation to a dose response may not be possible. Therefore, we urge caution in this interpretation, particularly until these results can be expanded and validated in a larger population.

Evidence from an autopsy study of adults has suggested strong correlations between levels of total Hg in toenails and MeHg levels in blood or occipital cortex (Björkman et al. 2007), suggesting that toenails are relevant biomarkers. Because of slow growth of toenails, toenail Hg likely reflects exposures in the past 3–5 months (Goullé et al. 2009). Thus, infant toenail Hg likely reflects prenatal exposures occurring over most of pregnancy. We note that toenail Hg observed in this cohort falls within toenail Hg reference ranges (Goullé et al. 2009), suggesting we are likely examining common, low-level variation in exposure and associations with methylation, which potentially could contribute to later developmental deficiencies. An important future direction will be investigating potential postnatal epigenetic × environment interactions in high-risk profile children in addition to confirming these findings in additional cohorts.

Limitations to this study include undetermined Hg exposure sources, infant toenail Hg as a proxy for prenatal exposure, use of term placentas, a relatively small sample size (including *n* = 16 high-risk NNNS profile infants), independent sample sets for Hg and neurobehavior analyses, which limits our ability to examine direct relationships between them, and a large proportion of samples falling below the limit of detection. Future analyses may benefit from examining, in larger data sets with Hg and NNNS data, whether high-risk profile infants were also exposed to more Hg. Since *EMID2* methylation has not been associated with Hg or neurodevelopment, and its placental function is unknown, it is unclear whether hypo- or hypermethylation with high-risk profile is expected, and future mechanistic and epidemiologic studies should investigate this.

## Conclusions

This study provides evidence for a potential role for placental epigenetic alterations as a mechanism linking Hg exposure and adverse infant neurodevelopment, and specifically a role for *EMID2*. This suggests possible associations between prenatal Hg exposure, placental methylation changes, and the developmental origins of mental/behavioral and physical health and disease.

## Supplemental Material

(1.5 MB) PDF
