# Supplementary material for: Placental DNA Methylation Related to Both Infant Toenail Mercury and Adverse Neurobehavioral Outcomes
Source: Environ Health Perspect. 2015 Mar 6;123(7):723–9. doi: 10.1289/ehp.1408561 (PMC4492267; doi:10.1289/ehp.1408561)

Environ Health Perspect
doi: 10.1289/ehp.1408561

Note to Readers: *EHP* strives to ensure that all journal content is accessible to all readers. However, some figures and Supplemental Material published in *EHP* articles may not conform to 508 standards due to the complexity of the information being presented. If you need assistance accessing journal content, please contact ehp508@niehs.nih.gov. Our staff will work with you to assess and meet your accessibility needs within 3 working days.

Supplemental Material

Placental DNA Methylation Related to Both Infant Toenail Mercury and Adverse Neurobehavioral Outcomes

Jennifer Z.J. Maccani, Devin C. Koestler, Barry Lester, E. Andrés Houseman, David A. Armstrong, Karl T. Kelsey, and Carmen J. Marsit

Table of Contents

Table S1. Mean and standard deviation of 13 NNNS scores in infants with non-high-risk vs high-risk NNNS profiles.

Table S2. Mean ß values and p-values of 339 CpG loci associated with infant toenail Hg (omnibus p-value=0.017; delta-β>0.125 between any two Hg tertiles). "NA" indicates that a locus does not fall within a defined gene region according to Illumina annotation data.

Figure S1. Heatmap demonstrating methylation in an independent set of 151 placental samples at 339 Hg-associated loci. Placental samples are in columns; rows are 339 loci. High-risk (red) and low-risk (blue) neurobehavioral profiles are color-coded above figure.

Figure S2. Plot of placental *EMID2* methylation at 6 CpG loci significantly associated with both infant toenail Hg tertile and high-risk neurobehavioral profile and placental *EMID2* gene expression: A) cg13267931, r= -0.40; B) cg27179533, r= -0.36; C) cg14874750, r= -0.39; D) cg23424003, r= -0.33; E) cg27528510, r=-0.37; F) cg14048874, r= -0.45.

Table S1. Mean and standard deviation of 13 NNNS scores in infants with non-high-risk vs high-risk NNNS profiles.

Mean (±SD)	Infants with Non-High-Risk NNNS Profiles (n=135)	Infants with High-Risk NNNS Profile (n=16)	p-Value
Habituation[a]	7.2 (± 1.6)	7.5 (± 0.7)	0.37
Attention [b]	3.8 (± 1.1)	3.4 (± 1.2)	0.34
Handling	0.4 (± 0.2)	0.6 (± 0.2)	0.0003
Quality of movement	4.1 (± 0.6)	3.0 (± 0.5)	6.33e-8
Regulation [c]	4.8 (± 0.8)	3.5 (± 0.5)	2.71e-9
Non-optimal reflexes	6.3 (± 2.0)	5.6 (± 3.0)	0.34
Asymmetric reflexes	1.9 (± 1.4)	1.8 (± 1.3)	0.77
Stress/abstinence	0.2 (± 0.1)	0.3 (± 0.1)	0.0009
Arousal	4.0 (± 0.8)	5.3 (± 0.4)	1.03e-11
Hypertonicity	0.3 (± 0.6)	1.2 (± 1.3)	0.02
Hypotonicity	0.7 (± 1.0)	0.6 (± 1.2)	0.73
Excitability	4.4 (± 2.5)	10.3 (± 1.2)	<2.2e-16
Lethargy	7.0 (± 2.4)	5.0 (± 2.4)	0.005

[a]60 samples missing habituation data; 53 non-high-risk samples, 7 high-risk samples. [b]16 samples missing attention data; 14 non-high-risk samples, 2 high-risk samples. [c]1 non-high-risk sample missing regulation data.

Table S2. Mean ß values and p-values of 339 CpG loci associated with infant toenail Hg (omnibus p-value=0.017; delta-β>0.125 between any two Hg tertiles). "NA" indicates that a locus does not fall within a defined gene region according to Illumina annotation data.

Illumina CpG Designation	Illumina Gene Symbol Annotation	p-Value for Association with Infant Toenail Hg Tertile	p-Value for Association with High Risk NNNS Profile	Mean ß-Value for Low Infant Toenail Hg Tertile (Referent)	Mean ß-Value for Medium Infant Toenail Hg Tertile	Mean ß-Value for High Infant Toenail Hg Tertile
cg09415955	CASZ1	3.9E-07	0.92	0.6311	0.5325	0.5042
cg00054702	LOC389332	2.1E-05	0.96	0.1879	0.3792	0.3443
cg02124514	LOC389332	1.9E-05	0.99	0.1719	0.3598	0.3348
cg06680397	LOC389332	5.4E-05	0.80	0.1127	0.2751	0.2550
cg09142848	NA	1.2E-05	0.17	0.4607	0.5990	0.5445
cg10454766	C11orf87	3.1E-05	0.47	0.2570	0.3966	0.3855
cg12195446	IRS2	4.7E-05	0.092	0.6218	0.4243	0.8543
cg21158476	LOC389332	5.2E-05	0.97	0.1063	0.2499	0.2226
cg21852408	C11orf87	1.8E-05	0.76	0.3703	0.5136	0.4987
cg05430997	NA	5.0E-05	0.95	0.6027	0.5561	0.6946
cg06430688	NA	1.4E-05	0.99	0.6778	0.6959	0.4826
cg14048874	EMID2	3.8E-05	0.0023	0.2264	0.2002	0.3416
cg19190016	NA	1.5E-05	0.67	0.4176	0.3562	0.2899
cg19497031	POU4F1	3.9E-05	0.28	0.0610	0.1298	0.2007
cg23119063	NA	1.4E-05	0.87	0.7726	0.7012	0.6268
cg22728830	NA	5.7E-05	0.21	0.4897	0.4477	0.3499
cg00146240	NA	6.4E-05	0.66	0.7624	0.4812	0.5847
cg04861929	C11orf87	8.0E-05	0.51	0.2831	0.3962	0.4118
cg05704942	MRTO4	0.00013	0.99	0.2033	0.4288	0.3943
cg05956126	LOC389332	0.00013	0.92	0.1260	0.2712	0.2512
cg07374961	NA	0.00011	0.70	0.4750	0.6225	0.5429
cg21422164	RASA3	0.00013	0.96	0.4465	0.5906	0.5149
cg13267931	EMID2	0.00014	8.3E-06	0.2602	0.2475	0.3818
cg02511231	TLX3	0.00013	0.99	0.4056	0.3100	0.2767
cg02673015	TP53TG1; CROT	0.00013	0.99	0.2233	0.2390	0.3527
cg07180307	C11orf87	0.00012	0.090	0.4450	0.5678	0.5745
cg06719900	C11orf87; C11orf87	0.00017	0.85	0.2754	0.4332	0.4515
cg15892115	ONECUT2	0.00016	0.97	0.2638	0.2622	0.4102
cg08768621	ONECUT2	0.00021	0.99	0.3477	0.3306	0.4574
cg09696939	BICC1	0.00019	0.43	0.3399	0.2621	0.2110
cg12969170	TP53TG1; CROT	0.00021	0.79	0.1662	0.1936	0.3016
cg12288267	NA	0.00022	0.16	0.5453	0.5920	0.4320

Illumina CpG Designation	Illumina Gene Symbol Annotation	p-Value for Association with Infant Toenail Hg Tertile	p-Value for Association with High Risk NNNS Profile	Mean ß-Value for Low Infant Toenail Hg Tertile (Referent)	Mean ß-Value for Medium Infant Toenail Hg Tertile	Mean ß-Value for High Infant Toenail Hg Tertile
cg05986417	NA	0.00025	0.19	0.4955	0.6680	0.5920
cg16063666	TP53TG1; CROT	0.00028	0.90	0.2234	0.2455	0.4053
cg05454501	ZBED3	0.00030	0.48	0.0781	0.2150	0.2657
cg13965724	P4HTM	0.00030	0.34	0.0312	0.1714	0.0739
cg09856068	FGF18	0.00039	0.12	0.3776	0.5570	0.4847
cg24642825	NA	0.00039	0.94	0.1708	0.3643	0.2318
cg03494795	ABL1	0.00035	0.62	0.2264	0.1224	0.2688
cg11464064	ABL1	0.00037	0.95	0.1927	0.0959	0.2380
cg03517284	NA	0.00040	0.92	0.2852	0.3670	0.4198
cg27179533	EMID2	0.00043	5.5E-05	0.3136	0.2811	0.4263
cg11144103	PTRF	0.00053	0.98	0.4191	0.2436	0.4795
cg13225272	NA	0.00058	0.19	0.7069	0.5499	0.6449
cg24900563	NA	0.00051	0.45	0.7672	0.6321	0.7386
rs4742386	NA	0.00058	0.85	0.7273	0.5202	0.4002
cg10288784	ABL1	0.00051	0.92	0.1564	0.0629	0.2020
cg14995921	SNORA15; CCT6A	0.00057	0.21	0.7592	0.6499	0.8003
cg11199014	LRFN1	0.00062	0.093	0.2460	0.2690	0.3955
cg20174000	ABL1	0.00068	0.90	0.2264	0.1427	0.2734
rs939290	NA	0.00076	0.81	0.7571	0.4248	0.5393
cg17339147	WNT2	0.00078	0.64	0.1570	0.1247	0.2514
cg27528510	EMID2	0.00084	9.0E-05	0.3664	0.3410	0.4853
cg00695177	NA	0.00086	0.18	0.6461	0.7235	0.8139
cg15436096	GPR135	0.00089	0.85	0.0431	0.1292	0.2411
cg03920544	NA	0.00099	0.24	0.5687	0.6052	0.4600
cg02720091	CASZ1	0.00097	0.30	0.5998	0.5185	0.4570
cg21882477	STK32A	0.00098	0.71	0.2560	0.2679	0.3819
cg16100355	MCF2L	0.0014	0.13	0.6194	0.7446	0.6441
cg19587838	NA	0.0012	0.25	0.4959	0.6267	0.5427
cg21864016	C14orf132	0.0013	0.97	0.8089	0.6453	0.7325
cg04032226	PYY;NAGS	0.0013	0.96	0.3106	0.3414	0.4726
cg07820332	NA	0.0014	0.74	0.6210	0.6629	0.5196
cg14874750	EMID2	0.0013	6.1E-05	0.3214	0.2922	0.4311
cg06183338	COL23A1	0.0013	0.60	0.3361	0.4324	0.4890
cg17163751	H2AFY2	0.0014	0.75	0.1354	0.1773	0.2695
cg23448505	HKR1	0.0016	0.26	0.3249	0.1923	0.2667
rs133860	NA	0.0016	0.99	0.5205	0.7887	0.7287
cg02090762	FSCN2	0.0016	0.99	0.3766	0.3573	0.4922
cg01393340	CD83	0.0018	0.98	0.4472	0.2979	0.3291
cg06711418	MT2A	0.0029	0.048	0.3163	0.1892	0.4524

Illumina CpG Designation	Illumina Gene Symbol Annotation	p-Value for Association with Infant Toenail Hg Tertile	p-Value for Association with High Risk NNNS Profile	Mean ß-Value for Low Infant Toenail Hg Tertile (Referent)	Mean ß-Value for Medium Infant Toenail Hg Tertile	Mean ß-Value for High Infant Toenail Hg Tertile
cg13133387	NA	0.0030	0.99	0.6845	0.8205	0.7823
cg13506281	MTUS2	0.0021	0.84	0.6911	0.5162	0.5153
cg13755866	NA	0.0024	0.13	0.4682	0.6041	0.5807
cg18391209	CAPN8	0.0021	0.40	0.6716	0.4133	0.5169
cg20919942	SSH2	0.0019	0.032	0.0659	0.1995	0.1463
cg26734888	HKR1	0.0026	0.17	0.2327	0.0909	0.1517
rs1416770	NA	0.0020	0.22	0.3147	0.5497	0.4305
cg09088988	STK32A	0.0028	0.079	0.2359	0.2179	0.3945
cg13590055	LOC100130522; PARD6G	0.0021	0.84	0.6616	0.6094	0.7359
cg16024904	B3GNT3	0.0023	0.60	0.2946	0.2934	0.1661
cg23424003	EMID2	0.0026	7.3E-05	0.4881	0.4476	0.5888
cg26402828	PCK2	0.0030	0.46	0.4709	0.3980	0.5288
cg27256423	NA	0.0027	0.95	0.7238	0.6532	0.7864
cg27331524	WNT2	0.0028	0.74	0.1308	0.0899	0.2345
cg00002033	LRFN1	0.0026	0.12	0.1010	0.1433	0.2457
cg00695416	CBR1	0.0028	0.045	0.3480	0.3890	0.5059
cg02096887	CASZ1	0.0026	0.071	0.6525	0.5708	0.4888
cg03841977	CBR1	0.0028	0.012	0.2868	0.3408	0.4412
cg08475953	COL23A1	0.0022	0.94	0.2436	0.3611	0.4579
cg14562786	FAM75A3	0.0030	0.55	0.6323	0.6494	0.7647
cg16657538	ZNF397OS	0.0028	0.010	0.5463	0.4826	0.4210
cg17240976	HDLBP	0.0023	0.73	0.4123	0.4916	0.5708
cg17882660	ZIC2	0.0026	0.58	0.1604	0.2504	0.2915
cg20606489	NA	0.0019	0.088	0.8170	0.7914	0.6765
cg23169584	EXOC4	0.0027	0.97	0.7152	0.7352	0.8513
cg27307781	CBR1	0.0025	0.085	0.3141	0.3784	0.5024
cg22244940	MMP17	0.0031	0.044	0.5573	0.6940	0.4970
cg24651215	NA	0.0032	0.094	0.6210	0.5590	0.6924
cg05280698	HKR1	0.0035	0.28	0.2259	0.0731	0.1389
cg24834889	HKR1	0.0035	0.32	0.2599	0.1278	0.1890
cg07697895	WNT2	0.0035	0.30	0.1309	0.0747	0.2489
cg12451631	RASGEF1A	0.0035	0.66	0.2416	0.3394	0.4089
cg07015190	RPS6KA2	0.0037	0.098	0.2399	0.3947	0.2603
cg13687570	HKR1	0.0037	0.19	0.2743	0.1292	0.1872
cg14166009	HKR1	0.0037	0.19	0.2667	0.1345	0.1954
cg03315469	NA	0.0042	0.33	0.4510	0.6101	0.5327
cg24385580	SSH2	0.0042	0.072	0.0688	0.1954	0.1441
cg03224005	NA	0.0041	0.29	0.5711	0.5145	0.3894
cg04517722	B3GNT3	0.0041	0.89	0.2899	0.2976	0.1631

Illumina CpG Designation	Illumina Gene Symbol Annotation	p-Value for Association with Infant Toenail Hg Tertile	p-Value for Association with High Risk NNNS Profile	Mean ß-Value for Low Infant Toenail Hg Tertile (Referent)	Mean ß-Value for Medium Infant Toenail Hg Tertile	Mean ß-Value for High Infant Toenail Hg Tertile
cg07229186	MT1M	0.0041	0.99	0.3300	0.3939	0.2687
cg26166804	H2AFY2	0.0045	0.96	0.1933	0.2252	0.3565
cg22655988	CRMP1	0.0046	0.26	0.2022	0.3596	0.2802
cg12024906	HKR1	0.0048	0.28	0.2924	0.1210	0.2025
cg12948621	HKR1	0.0050	0.17	0.3221	0.1584	0.2537
cg19080354	ATHL1	0.0050	0.75	0.4473	0.3090	0.4037
cg16322262	GPR135	0.0048	0.57	0.1600	0.2088	0.2948
cg16662267	CBR1	0.0050	0.049	0.1957	0.2569	0.3456
cg21893185	GPR135	0.0049	0.82	0.0381	0.1342	0.2224
cg05237503	FBXO3	0.0052	0.99	0.6767	0.4644	0.5278
cg08858160	NA	0.0053	0.83	0.4897	0.6220	0.5267
cg10320659	NA	0.0054	0.74	0.2884	0.4184	0.3760
cg26910511	LRFN1	0.0053	0.22	0.2166	0.2153	0.3555
cg22455450	ZNF808	0.0054	0.34	0.2964	0.3390	0.4231
cg10165894	NA	0.0055	0.86	0.3434	0.4511	0.4709
cg07686394	NA	0.0056	0.19	0.7276	0.5698	0.5779
cg05712748	NA	0.0059	0.10	0.7463	0.6059	0.5655
cg00321709	CYP2E1	0.0059	0.91	0.3876	0.3066	0.4417
cg16744531	B3GNT3	0.0059	0.60	0.3594	0.3680	0.2114
cg12386614	NA	0.0060	0.062	0.6313	0.6844	0.7794
cg10014563	GPR135	0.0066	0.99	0.1475	0.2055	0.2889
cg05115233	RASA3	0.0070	0.95	0.5088	0.6467	0.5695
cg16891431	TCERG1L	0.0068	0.88	0.5291	0.3932	0.4436
cg21149357	NA	0.0071	0.13	0.5176	0.3922	0.5751
cg11009736	MARCO	0.0071	0.92	0.5728	0.4702	0.4318
cg15210622	PPP2R3A	0.0072	0.064	0.5278	0.6297	0.6612
cg22821834	GPR135	0.0067	0.94	0.1368	0.2065	0.2972
cg15165122	ANKRD53	0.0077	0.99	0.2914	0.4259	0.3491
cg15351736	DIXDC1	0.0077	0.21	0.3016	0.1617	0.2184
cg00243527	LIF	0.0077	0.25	0.6016	0.6153	0.7595
cg27467876	SLC39A14	0.0074	0.42	0.7175	0.6616	0.5346
cg22848598	ADAM32	0.0075	0.87	0.4304	0.3193	0.2763
cg15425276	ACTN1	0.0083	0.98	0.7486	0.6233	0.6682
cg08422420	SDHAP3	0.0085	0.62	0.3187	0.1930	0.3915
cg03022609	DIXDC1	0.0092	0.20	0.2776	0.1452	0.2066
cg08778598	SDHAP3	0.0092	0.66	0.3490	0.2036	0.4345
cg11484348	DEFB116	0.0094	0.028	0.4903	0.6161	0.5534
cg09310644	ZFP90	0.0091	0.76	0.4935	0.3954	0.5276
cg10470368	NA	0.0094	0.0075	0.6863	0.6007	0.7413

Illumina CpG Designation	Illumina Gene Symbol Annotation	p-Value for Association with Infant Toenail Hg Tertile	p-Value for Association with High Risk NNNS Profile	Mean ß-Value for Low Infant Toenail Hg Tertile (Referent)	Mean ß-Value for Medium Infant Toenail Hg Tertile	Mean ß-Value for High Infant Toenail Hg Tertile
cg02636041	RASGEF1A	0.0092	0.32	0.2299	0.3200	0.3673
cg04186360	GPR135	0.0088	0.99	0.0382	0.0966	0.1674
cg06721546	SDHAP3	0.0095	0.30	0.3205	0.2107	0.3724
cg10485664	FEZF1	0.010	0.13	0.1754	0.3530	0.2249
cg17951978	HSD3B2	0.010	0.090	0.8166	0.6787	0.7715
cg10172783	NAGS; PYY	0.010	0.87	0.2578	0.3123	0.3878
cg09859398	GPR135	0.011	0.94	0.0400	0.1247	0.2291
cg13401703	TTC23	0.012	0.029	0.4096	0.2715	0.3928
cg20206437	RPS6KA2	0.012	0.25	0.2760	0.4055	0.3026
cg24139169	DIXDC1	0.012	0.37	0.3286	0.1901	0.2596
cg25385940	TTC23	0.012	0.0059	0.4391	0.3012	0.4072
cg11093548	CREB5	0.012	0.19	0.2433	0.1745	0.3358
cg19653246	MIR654; MIR376B; MIR376A1; MIR376A2; MIR376C	0.012	0.13	0.5085	0.5966	0.6733
cg04990378	C20orf166; MIR133A2	0.012	0.76	0.5715	0.4245	0.4697
cg27149073	SDHAP3	0.013	0.35	0.3945	0.2649	0.4597
cg10441365	SAR1B	0.013	0.45	0.0964	0.1137	0.2639
cg21488538	CNGA3	0.013	0.86	0.3399	0.4236	0.2986
cg10512745	DMRTA2	0.014	0.49	0.1849	0.3128	0.2012
cg24603464	MLPH	0.014	0.99	0.3574	0.4321	0.3062
cg05114178	OR5R1; OR8U8	0.015	0.98	0.4521	0.5772	0.4179
cg17974166	NA	0.015	0.030	0.4457	0.5709	0.5040
cg03976877	VIPR2	0.015	0.83	0.2981	0.4093	0.2647
cg12556569	APOA5	0.015	0.73	0.2143	0.1852	0.3560
cg00543972	DIXDC1	0.016	0.73	0.3027	0.1483	0.2114
cg03084184	JAKMIP3	0.017	0.99	0.4738	0.6098	0.4368
cg06520095	NA	0.017	0.72	0.5919	0.7537	0.5850
rs2208123	NA	0.017	0.99	0.4596	0.6734	0.5167
cg20250269	NA	0.017	0.97	0.4939	0.5717	0.3882
cg24960960	SDHAP3	0.017	0.72	0.2659	0.1709	0.3482
cg15652532	LCLAT1; LCLAT1	0.017	0.90	0.2461	0.3144	0.3841
cg23136139	RASGEF1A	0.017	0.77	0.3617	0.4472	0.4935
cg27378537	SDHAP3	0.018	0.47	0.3742	0.2422	0.4233
cg21931717	SDHAP3	0.018	0.71	0.2647	0.1702	0.3383
cg05593887	MAGI2	0.018	0.99	0.5444	0.5860	0.6770
cg02113055	NA	0.019	0.62	0.3803	0.6817	0.4398
cg08900396	RPH3AL	0.019	0.10	0.5842	0.7139	0.7854
cg25885322	DIXDC1	0.019	0.26	0.3026	0.1658	0.2351

Illumina CpG Designation	Illumina Gene Symbol Annotation	p-Value for Association with Infant Toenail Hg Tertile	p-Value for Association with High Risk NNNS Profile	Mean ß-Value for Low Infant Toenail Hg Tertile (Referent)	Mean ß-Value for Medium Infant Toenail Hg Tertile	Mean ß-Value for High Infant Toenail Hg Tertile
cg25465065	PMF1	0.020	0.99	0.3851	0.4422	0.6057
cg21543270	PACS2	0.020	0.40	0.5670	0.6689	0.7057
cg21558508	SAR1B	0.020	0.89	0.0375	0.0566	0.1695
cg05714155	NA	0.022	0.54	0.6045	0.4661	0.5104
cg16511983	NA	0.022	0.34	0.2933	0.4133	0.2582
cg17128947	CPLX1	0.022	0.0054	0.6007	0.4302	0.4833
cg12624040	B3GALT1	0.022	0.020	0.6396	0.7331	0.6001
cg12466610	MOSC2	0.023	0.73	0.2007	0.1890	0.4350
cg02589828	SAR1B	0.024	0.35	0.1308	0.1560	0.2600
cg24714905	ZIC2	0.024	0.89	0.2925	0.3706	0.4186
cg14056849	LOC169834	0.026	0.84	0.4439	0.3456	0.3152
cg00303341	NA	0.027	0.92	0.7786	0.8191	0.6924
cg26472636	SDHAP3	0.029	0.40	0.4083	0.2616	0.4876
cg18828306	USH1C	0.029	0.89	0.5526	0.4857	0.6460
cg17662493	SMC1B	0.029	0.49	0.5369	0.6454	0.7041
cg00332745	NOL3	0.031	0.85	0.2640	0.2023	0.3437
cg05134500	MFI2	0.031	0.56	0.1443	0.2271	0.1016
cg07875818	PLXNA4	0.031	0.51	0.3249	0.2666	0.4337
cg23490161	SDHA; CCDC127	0.031	0.85	0.2772	0.1634	0.3155
cg15385386	SAR1B	0.031	0.66	0.0951	0.1166	0.2311
cg23892028	NA	0.031	0.99	0.4691	0.3832	0.3165
cg01356752	NA	0.032	0.64	0.6305	0.7111	0.7755
rs739259	NA	0.035	0.10	0.3100	0.4612	0.4155
cg12031275	NA	0.035	0.67	0.7346	0.7267	0.5979
cg00980980	NA	0.035	0.99	0.5547	0.6787	0.7121
rs2125573	NA	0.036	0.99	0.7329	0.5373	0.5530
cg16744961	CLECL1	0.037	0.93	0.5067	0.5895	0.6572
cg22664298	ADAMTS19	0.039	0.075	0.2887	0.1542	0.1658
cg27452255	NA	0.039	0.27	0.7364	0.5537	0.7113
cg16519587	NA	0.039	0.30	0.3560	0.2804	0.4060
cg20381372	LOC100134317	0.039	0.96	0.6251	0.6904	0.7805
cg00689685	AGPAT1	0.041	0.31	0.5495	0.6326	0.7122
cg08049519	NA	0.043	0.021	0.3551	0.2713	0.5108
cg03349922	H2AFY2	0.044	0.93	0.1569	0.2019	0.2852
cg01336390	NA	0.045	0.75	0.2105	0.3622	0.2885
cg13697578	NA	0.045	0.92	0.5953	0.6190	0.4937
cg14175932	NA	0.046	2.8E-05	0.6741	0.5552	0.7104
cg03358735	ADAMTS17	0.046	0.78	0.8455	0.7987	0.6949
cg12454169	LCLAT1	0.047	0.99	0.1831	0.2716	0.3224

Illumina CpG Designation	Illumina Gene Symbol Annotation	p-Value for Association with Infant Toenail Hg Tertile	p-Value for Association with High Risk NNNS Profile	Mean ß-Value for Low Infant Toenail Hg Tertile (Referent)	Mean ß-Value for Medium Infant Toenail Hg Tertile	Mean ß-Value for High Infant Toenail Hg Tertile
rs9363764	NA	0.048	0.016	0.6632	0.4439	0.4917
cg14264023	COLEC11	0.048	0.98	0.4861	0.4507	0.3341
cg24836826	NCOR2	0.049	0.94	0.6887	0.7709	0.8368
cg17635970	HHLA1	0.049	0.45	0.6469	0.5036	0.7007
cg26531700	NA	0.050	0.98	0.4140	0.3454	0.4731
cg13251750	SERPINA9	0.052	0.046	0.4595	0.3507	0.3286
cg14317384	NA	0.053	0.79	0.7288	0.6230	0.5987
cg18260973	SAR1B	0.054	0.40	0.0542	0.0809	0.2278
rs4331560	NA	0.056	0.51	0.5485	0.4149	0.5033
cg02658043	NRBP2	0.057	0.99	0.6361	0.5033	0.5934
cg18237047	NA	0.057	0.73	0.6641	0.5147	0.5885
cg08849813	SERGEF	0.059	0.30	0.6289	0.7286	0.5543
cg25420101	WDR41	0.060	0.95	0.4935	0.3438	0.4036
cg23318063	NA	0.061	0.87	0.3075	0.2529	0.3804
rs966367	NA	0.064	0.86	0.5019	0.2892	0.4370
cg11497957	ZNF572	0.069	0.13	0.3429	0.2062	0.2477
cg19245335	PRDM15	0.068	0.95	0.7400	0.5900	0.5349
cg01127608	LMX1B	0.069	0.37	0.5105	0.5975	0.4577
cg05062612	WSCD2	0.070	0.99	0.3202	0.4616	0.3991
cg09481537	NA	0.071	0.57	0.4696	0.5266	0.3696
cg10156366	NA	0.071	0.039	0.2463	0.3116	0.1755
cg27591450	NA	0.072	0.99	0.3055	0.2994	0.1789
cg05900567	NA	0.077	0.043	0.4032	0.4542	0.5863
cg19726179	H2AFY2	0.080	0.80	0.1433	0.1841	0.2869
rs264581	NA	0.081	0.36	0.2716	0.4195	0.4597
cg05279330	NA	0.083	0.20	0.6516	0.5806	0.7157
rs1941955	NA	0.087	0.79	0.5935	0.6044	0.8018
rs10774834	NA	0.090	0.54	0.6052	0.7993	0.5855
cg01948148	SAR1B	0.091	0.77	0.0742	0.0855	0.2234
cg17323488	NA	0.091	0.58	0.4136	0.3826	0.2885
cg13236378	WSCD2	0.10	0.99	0.3095	0.4413	0.3780
cg14554788	C3orf26	0.11	0.75	0.8363	0.7110	0.8426
cg15170605	ADD2	0.11	0.40	0.2480	0.2377	0.4482
cg05519582	GRAMD4	0.11	0.58	0.5688	0.6391	0.7130
cg08024264	SCARNA16; C17orf86	0.11	0.79	0.4630	0.3074	0.3248
cg26293310	SAR1B	0.11	0.49	0.0591	0.0785	0.2373
cg12894709	PURA	0.12	0.98	0.4358	0.5740	0.4829
cg15845365	MYO10	0.12	0.19	0.2852	0.1516	0.2836
cg20673830	NA	0.12	0.62	0.4777	0.4224	0.5999

Illumina CpG Designation	Illumina Gene Symbol Annotation	p-Value for Association with Infant Toenail Hg Tertile	p-Value for Association with High Risk NNNS Profile	Mean ß-Value for Low Infant Toenail Hg Tertile (Referent)	Mean ß-Value for Medium Infant Toenail Hg Tertile	Mean ß-Value for High Infant Toenail Hg Tertile
cg27230769	LOC285830	0.12	0.83	0.5095	0.4939	0.6819
cg13275129	MAML2	0.12	0.80	0.6344	0.7412	0.7726
cg20187719	LOC285375	0.12	0.38	0.3162	0.2591	0.1697
cg06417478	HOOK2	0.12	0.82	0.3427	0.5011	0.3551
rs798149	NA	0.12	0.27	0.5868	0.4762	0.4159
cg04194432	NA	0.12	0.81	0.4056	0.2480	0.3826
rs715359	NA	0.13	0.88	0.7906	0.7184	0.5900
cg23160717	PURA	0.13	0.87	0.4395	0.5683	0.4794
cg07021532	ZFP2	0.13	0.72	0.2070	0.3495	0.3775
rs1510480	NA	0.13	0.39	0.3765	0.5571	0.4669
rs2385226	NA	0.13	0.93	0.5507	0.3302	0.4722
rs6982811	NA	0.14	0.32	0.5042	0.3022	0.4201
cg03384579	ADD2	0.14	0.45	0.1406	0.1370	0.2788
rs951295	NA	0.15	0.94	0.4017	0.2953	0.4416
rs5936512	NA	0.15	0.32	0.1812	0.3388	0.3164
cg02343823	ZNF300	0.15	0.28	0.5007	0.6298	0.4677
cg11593111	PURA	0.15	0.96	0.4493	0.5791	0.4859
cg12469381	CHN2	0.16	0.13	0.5052	0.4908	0.6716
cg24694833	NA	0.16	0.89	0.5718	0.5669	0.7444
cg18318704	SAR1B	0.17	0.52	0.0478	0.0636	0.1914
rs6546473	NA	0.18	0.92	0.3187	0.1223	0.2504
cg04453550	TNFRSF17	0.18	0.96	0.5815	0.5156	0.6935
cg25649515	NA	0.18	0.16	0.5326	0.5828	0.4472
rs654498	NA	0.18	0.31	0.6217	0.5416	0.6780
cg05971102	NA	0.19	0.83	0.4956	0.6279	0.5225
cg02546601	NA	0.19	0.72	0.6029	0.6777	0.5250
rs2857639	NA	0.20	0.24	0.5360	0.5245	0.3058
cg08603678	EIF3E	0.20	0.34	0.5358	0.7052	0.5806
cg13092806	NA	0.20	0.98	0.3146	0.3992	0.2340
cg05659187	ADD2	0.21	0.30	0.2206	0.1956	0.3540
cg13568515	JAK2	0.21	0.51	0.9237	0.7987	0.9311
cg04675542	ZNF300	0.21	0.35	0.4763	0.5849	0.4556
cg23427269	ADD2	0.21	0.21	0.2090	0.1748	0.3499
cg19863210	DENND3	0.22	0.96	0.2719	0.3985	0.3165
cg15454726	NA	0.22	0.094	0.1802	0.2461	0.3186
cg07501029	KIF26B	0.23	0.95	0.5647	0.3774	0.4905
cg20592836	TP53INP2	0.23	0.96	0.2940	0.4003	0.2660
cg18816122	PLEKHG4B	0.23	0.92	0.3944	0.4939	0.5513
cg21203249	SEMA4D	0.24	0.56	0.5604	0.4966	0.6472

Illumina CpG Designation	Illumina Gene Symbol Annotation	p-Value for Association with Infant Toenail Hg Tertile	p-Value for Association with High Risk NNNS Profile	Mean ß-Value for Low Infant Toenail Hg Tertile (Referent)	Mean ß-Value for Medium Infant Toenail Hg Tertile	Mean ß-Value for High Infant Toenail Hg Tertile
rs9292570	NA	0.26	0.50	0.6088	0.5916	0.4260
cg19783435	ARVCF	0.26	0.80	0.1670	0.2934	0.2097
cg22109827	NA	0.26	0.64	0.5077	0.5809	0.6521
cg18237551	ZNF300	0.26	0.57	0.4189	0.5450	0.3609
cg07258916	PLXNA4	0.27	0.49	0.1574	0.0553	0.1911
cg02907150	PCNX	0.28	0.99	0.5630	0.4569	0.6011
rs10033147	NA	0.28	0.99	0.5088	0.6533	0.6220
cg26864826	NA	0.28	0.90	0.4204	0.3237	0.2850
cg21913319	COL24A1	0.29	0.99	0.2864	0.1818	0.3246
cg12568536	NA	0.30	0.96	0.5203	0.3809	0.5326
cg11291313	ZNF300	0.30	0.53	0.4040	0.4960	0.3654
cg01891583	USP7	0.30	0.59	0.5369	0.5995	0.3765
cg24347663	ADD2	0.30	0.32	0.1928	0.1681	0.3234
cg03609614	ZNF300	0.33	0.063	0.2995	0.4193	0.2841
cg27639199	TMC3	0.34	0.47	0.2734	0.3809	0.3990
cg03988092	NA	0.35	0.99	0.1899	0.2053	0.3313
cg08857144	ADD2	0.36	0.24	0.2065	0.1846	0.3322
cg22851875	NA	0.37	0.56	0.5811	0.5948	0.4395
cg04462931	NA	0.38	0.24	0.6811	0.5663	0.7113
cg21164300	NA	0.39	0.15	0.3268	0.4501	0.2925
cg04610028	RAB11B	0.39	0.067	0.6429	0.5844	0.7462
rs1484127	NA	0.43	0.88	0.5191	0.7044	0.5806
cg10376408	SCUBE2	0.43	0.99	0.4842	0.4112	0.5436
rs5987737	NA	0.45	0.30	0.6220	0.4909	0.6358
rs13369115	NA	0.45	0.99	0.6057	0.7165	0.5307
cg03449867	OCA2	0.52	0.62	0.4201	0.3456	0.5356
cg00988148	NA	0.55	0.92	0.3586	0.2481	0.3980
cg21228005	ZNF300	0.55	0.097	0.4459	0.5307	0.4013
cg26846609	THUMPD1	0.56	0.97	0.4786	0.5349	0.6100
cg24844518	CYFIP2	0.58	0.027	0.5423	0.4271	0.5851
cg16043227	CCR6	0.62	0.087	0.5333	0.6000	0.4591
cg17138852	NA	0.62	0.98	0.3936	0.3040	0.4512
cg24506221	GSTM1	0.70	0.011	0.4747	0.3923	0.5269
rs3818562	NA	0.72	0.72	0.5568	0.4087	0.5327
cg20696478	ZNF175	0.85	0.15	0.2371	0.3065	0.1675
cg07319199	ANTXR1	0.89	0.047	0.6930	0.7818	0.6453

Figure S1. Heatmap demonstrating methylation in an independent set of 151 placental samples at 339 Hg-associated loci. Placental samples are in columns; rows are 339 loci. High-risk (red) and low-risk (blue) neurobehavioral profiles are color-coded above figure.

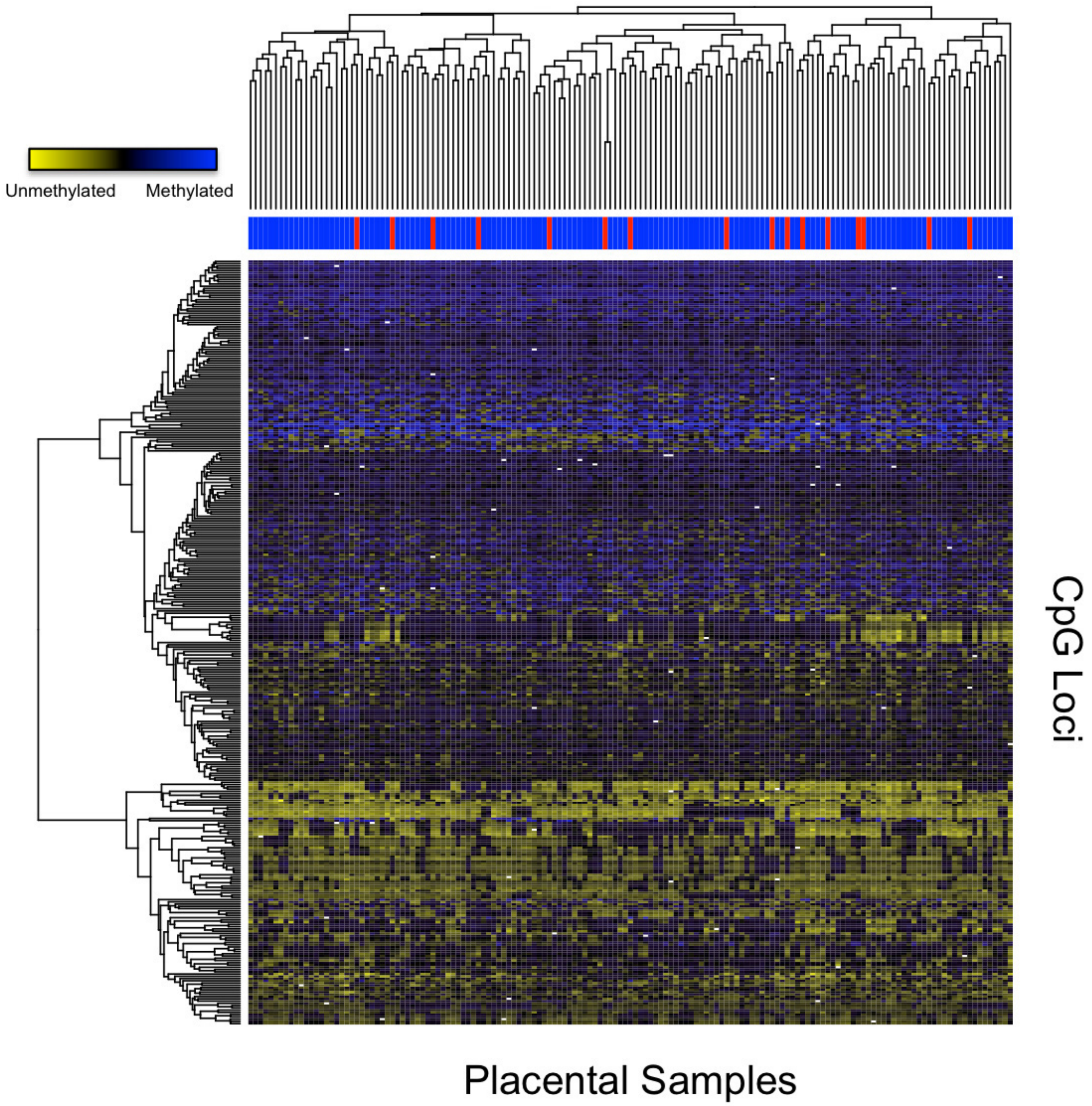

Figure S2. Plot of placental *EMID2* methylation at 6 CpG loci significantly associated with both infant toenail Hg tertile and high-risk neurobehavioral profile and placental *EMID2* gene expression: A) cg13267931, r= -0.40; B) cg27179533, r= -0.36; C) cg14874750, r= -0.39; D) cg23424003, r= -0.33; E) cg27528510, r=-0.37; F) cg14048874, r= -0.45.

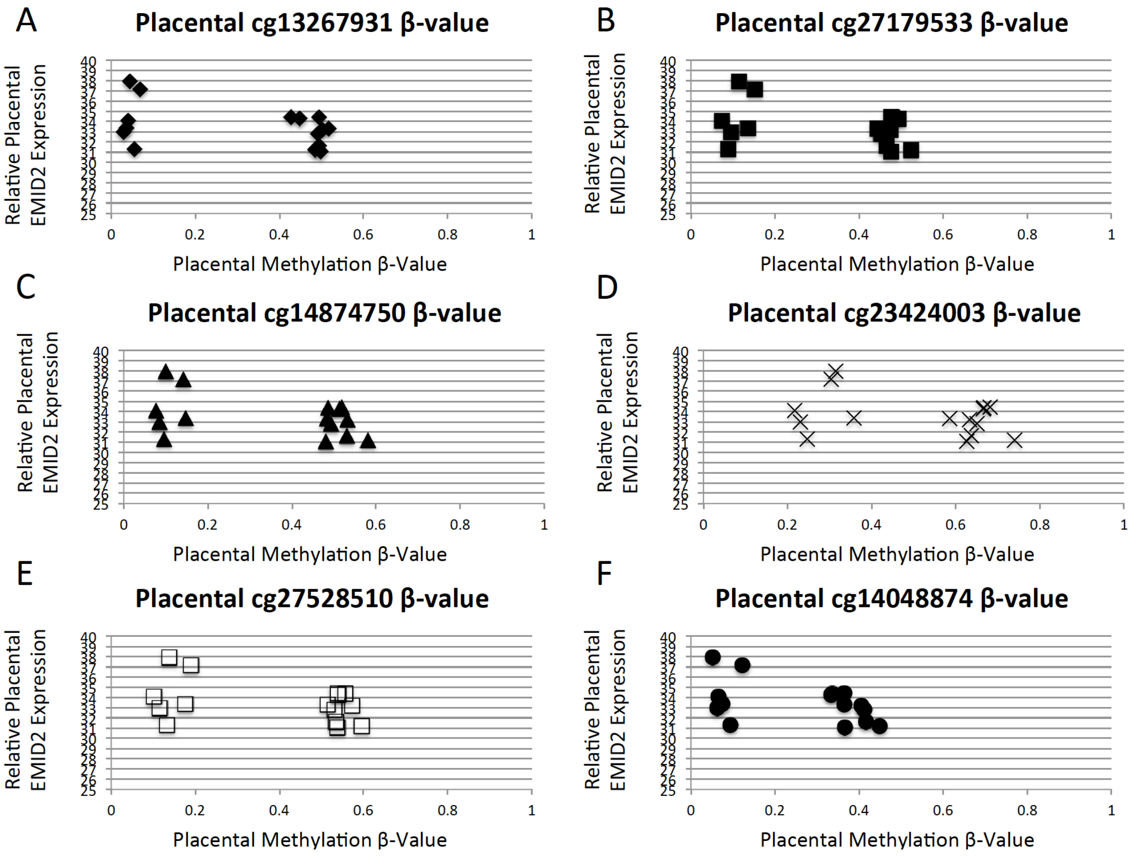

Supplement: (1.5 MB) PDF [file ehp.1408561.s001.acco.pdf]
